# Congenital Hypofibrinogenemia in a Neonate with a Novel Mutation in the *FGB* Gene

**DOI:** 10.3390/pediatric13010016

**Published:** 2021-03-01

**Authors:** Jun Shinozuka, Nobuo Okumura, Mayumi Nagasawa, Motokazu Nishikado, Sayaka Kadowaki, Itsuro Katsuda, Shinsaku Imashuku

**Affiliations:** 1 Uji-Tokushukai Medical Center, Department of Pediatrics, Uji, Kyoto 611-0041, Japan; jun_shino@hotmail.com (J.S.); chucan0629@gmail.com (M.N.); tekimokusai@yahoo.co.jp (M.N.); sayakadowaki@gmail.com (S.K.); 2 Department of Clinical Laboratory Investigation, Graduate School of Medicine, Shinshu University, Matsumoto, Nagano 390-8621, Japan; nobuoku@shinshu-u.ac.jp; 3 Department of Hematology, Fujita Health University, Toyoake, Aichi 470-1192, Japan; ikatsuda@fujita-hu.ac.jp; 4 Uji-Tokushukai Medical Center, Department of Laboratory Medicine, Uji, Kyoto 611-0041, Japan

**Keywords:** newborn, congenital hypofibrinogenemia, fibrinogen, blood coagulation disorders, gene mutation

## Abstract

Detection of severe hypofibrinogenemia (<50 mg/dL) in a neonate soon after birth is alarming because of the risk of hemorrhage. A female neonate was noted to be hypofibrinogenemic (<50 mg/dL) on day 0 of birth; she showed no thrombocytopenia/coagulopathy or hemorrhagic symptoms. Considering the possibility of afibrinogenemia, which may cause bleeding, fresh frozen plasma (FFP) was initiated twice a week to maintain her plasma fibrinogen level at 50–100 mg/dL. Thereafter, we found hypofibrinogenemia in her father and elder sister and plasma fibrinogen levels, determined by clot formation and immunological methods, showed similarly reduced values in both the neonate (proband) and her father. Based on a presumed diagnosis of congenital hypofibrinogenemia, sequencing of the fibrinogen genes was performed, revealing a novel heterozygous mutation of *FGB* (Genbank NG008833); a p.403Try>Stop. The neonate was treated with repeat FFP infusions until two months of age, when treatment was stopped because she remained asymptomatic.

## 1. Introduction

When markedly reduced fibrinogen (FIB) levels (<50 mg/dL) is noted in neonates, rapid differentiation of familial hemophagocytic lymphohistiocytosis [1] from rare congenital (hereditary) afibrinogenemia/hypofibrinogenemia [2,3,4] may be required. There are two types of hereditary FIB abnormalities: type I, quantitative FIB deficiencies (afibrinogenemia/hypofibrinogenemia) with autosomal recessive inheritance, and type II, qualitative FIB deficiencies (dysfibrinogenemia or hypodysfibrinogenemia), with autosomal dominant inheritance [3]. Congenital afibrinogenemia is usually caused by mutations in both the maternal and paternal copies of either the *FGA*, *FGB*, or *FGG* genes, while congenital hypofibrinogenemia is due to a disruptive mutation in only one of the two parental *FGA*, *FGB*, or *FGG* genes. In congenital dysfibrinogenemia, plasma FIB is a dysfunctional molecule, containing components generated from a mutated *FGA*, *FGB*, or *FGG* gene inherited from either parent [5]. Clinically, plasma FIB levels can be measured by clot formation (FIB activity) or immunological (FIB content as protein) assay methods. Plasma FIB protein levels are <10 mg/dL in afibrinogenemia, 50–150 mg/dL in hypofibrinogenemia, and >150 mg/dL in dysfibrinogenemia (in this disease, FIB activity is much lower). Congenital afibrinogenemia is generally associated with mild-to-severe bleeding, whereas hypofibrinogenemia is most often asymptomatic. Here, we report a female newborn with plasma FIB activity <50 mg/dL.

## 2. Case Report

The baby was born at 36 weeks of gestation by vaginal delivery from a G3P1 35-year-old mother. Birth weight was 2085 g. At birth, her Apgar score was 2/4/7, and she had severe asphyxia and was promptly intubated. Laboratory data (Table 1) showed that plasma FIB activity was extremely low (<50 mg/dL), but platelet count was normal and there was no coagulopathy. Other data were within normal limits.

We immediately started infusion of 10 mL/kg fresh frozen plasma (FFP), because of easier availability than fibrinogen concentrate. Considering the 3-day half-life of FIB, FFP infusion was performed twice per week. FIB levels once reached 101 mg/dL, but when infusion was given once per week, it returned to <50 mg/dL. Thereafter we continued FFP (once we employed fibrinogen concentrate) twice a week. Fortunately, a computed tomography scan of her head taken on day 3 showed mild left temporal cephalohematoma but was free from intracranial hemorrhage. On day 13, her brain MRI did not show any abnormal findings. During this period, we received information about her father, who had been hypofibrinogenemic since childhood and we found that her elder sister was also hypofibrinogenemic. Her mother had normal FIB levels. Using two methods (clot formation and immunological) to assay FIB, the patient (proband) had levels of 61 and 67 mg/dL and her father of 99 and 97 mg/dL, respectively. Thus, we diagnosed that three of this family members have congenital hypofibrinogenemia (Table 2).

**Figure 1:**
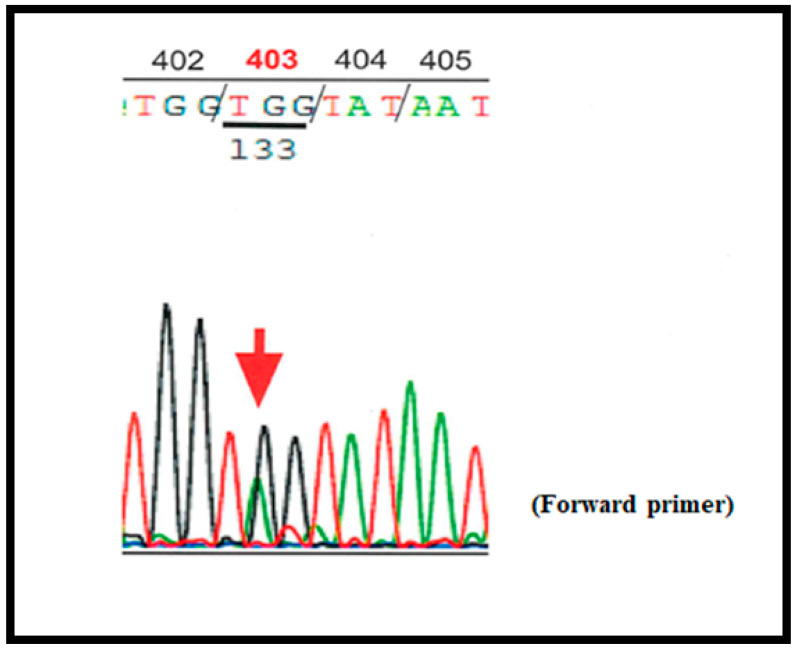
Chromatogram showing the heterozygous mutation in *FGB* (Genbank NG008833) encoding Bβ chain; p.403Trp (TGG) > Stop (TAG).

Thereafter, sequencing of gene encoding FIB was performed; genomic DNA extracted from whole blood cells was submitted for sequencing of all exons and exon-intron boundaries in the genes encoding Aα-, Bβ-, and γ-chain by Sanger method. Results revealed that the patient and her father had a heterozygous mutation in exon 8 of *FGB* (Genbank NG008833) Bβ p.403Try > Stop (amino acid residue number in mature protein; based on the current notation), which is a novel variant (Figure 1, Table 2). We continued FFP infusion followed by fibrinogen concentrate until 2 months after birth and then stopped. Plasma FIB levels have since been carefully monitored in the patient to prevent hemorrhage.

## 3. Discussion

FIB is a 340 kDa hexameric protein synthesized in the liver, which comprises three homologous peptide chains, Aα, Bβ, and γ; each polypeptide is encoded by a distinct gene, *FGA*, *FGB*, or *FGG*, respectively, and forms a cluster on human chromosome 4 (4q31–32) [6]. Causative mutations leading to congenital quantitative changes in fibrinogen are frequently clustered in *FGA*, which encodes the fibrinogen Aα chain [6]. Homozygous or compound heterozygous null mutations in *FGA* are the most common cause of afibrinogenemia, while heterozygosity of such mutations leads to hypofibrinogenemia [7]. Mutations of *FGB*, which encode the Bβ chain, are less common. Functionally, the Bβ chain is the rate-limiting factor in hepatic production of the fibrinogen hexamer. Our patient and her father had a novel *FGB* mutation, p.403Try > Stop. Experiments in CHO cells, to analyze the functional impact of this mutation, are in progress (Okumura N; in preparation). In the past, a p.Try403Leu mutation was reported at this site, as were others in adjacent (p.Try402 > Stop) and nearby (p.Try407 > Stop) amino acids in the Bβ chain, all of which were classified as causing hypofibrinogenemia [8].

A global survey of plasma FIB levels and hemorrhagic symptoms in 100 patients with congenital afibrinogenemia/hypofibrinogenemia (age range, 7 months–75 years) showed that 72 had plasma FIB levels <10 mg/dL, while 26 had levels of 10–50 mg/dL. Comparisons between these two groups demonstrated that bleeding in the central nervous system CNS/intra-retroperitoneal space, thrombosis, and miscarriage were found only in the former [9]. Evaluation of genotype/phenotype correlations by Chinni et al. demonstrated that four of six individuals with congenital hypofibrinogenemia caused by heterozygous mutations of *FGA*, *FGB*, or *FGG* were symptomatic [10]. In our case, when we first received a report of plasma FIB activity <50 mg/dL, we suspected afibrinogenemia and continued FFP; however, after confirmation of congenital hypofibrinogenemia in this family including her father and elder sister who have had no hemorrhagic symptoms, we stopped FFP/fibrinogen concentrate at 2 months of age.

Bleeding in the neonatal period generally results from thrombocytopenia, sepsis, or vitamin K deficiency. In cases of reduced fibrinogen, familial hemophagocytic lymphohistiocytosis [1] and other congenital bleeding disorders [2] should be considered as potential rare causes. Zhou et al. classified hospitalized neonates with hypofibrinogenemia (including acquired cases) into severe (FIB < 50 mg/dL), moderate (50–70 mg/dL), and mild (70–100 mg/dL) groups, based on their clinical characteristics. Severe hemorrhage may develop when hypofibrinogenemia co-occurs with either coagulopathies, thrombocytopenia, or both, and does not depend on the degree of hypofibrinogenemia alone [11]. In cases of neonatal congenital afibrinogenemia/hypofibrinogenemia, hemorrhagic symptoms such as antenatal intracranial bleeding and umbilical cord bleeding, have mostly been reported in afibrinogenemia [12,13,14,15,16] but rarely in hypofibrinogenemia [17,18]. Furthermore, in a report of 11 births from a mother with hypofibrinogenemia, five of twelve neonates were diagnosed with low levels of FIB in the 6 weeks after birth, in whom no bleeding episodes were described [19]. Fortunately, our case had no co-morbidities, such as thrombocytopenia; thus, she remained symptom-free though she had congenital hypofibrinogenemia. Based on our experience with this family, it is assumed that cases of congenital hypofibrinogenemia could be unrecognized or under diagnosed unless plasma FIB is determined, because hemorrhagic symptoms are scarce.

FIB replacement therapy, using FFP or fibrinogen concentrate, in doses adequate to achieve and maintain fibrinogen activity above 50–100 mg/dL, is generally effective in preventing or treating bleeding levels for non-surgical and obstetric purposes. However, in congenital hypofibrinogenemia, such measures need to be individualized. A risk of thromboembolic complications with or without replacement treatment was mentioned in cases of congenital afibrinogenemia [20,21].

In summary, we report here on a Japanese female newborn diagnosed with congenital hypofibrinogenemia with a novel mutation at the *FGB* gene (at exon 8, p.403Try > Stop). During the 2-month period after birth, her FIB levels were maintained at 50–100 mg/dL using FFP/fibrinogen concentrate. Fortunately, the patient did not develop any hemorrhagic episodes.

## Figures and Tables

**Table 1:** Laboratory data at birth.

**CBC**		**Coagulation Data**	
WBC (3000–8500)/µL	12,500	PT activity (80–100)%	42.8
Hb (11–16) g/dL	18.9	PT-INR (0.9–1.1)	1.62
MCV (83–100) fL	111.4	APTT (control) s	66.4 (27.3)
Reticulocyte (3–11)‰	53	Fibrinogen (200–400) mg/dL	<50
Platelet count (150 K–360 K)/µL	197 K	D-dimer (<1.0) µg/mL	2.7
**Biochemical data**			
CRP (<0.29) mg/dL	<0.01	Albumin (4.1–5.2) g/dL	3.1
AST (13–37) U/L	36	BUN (7.8–18.9) mg/dL	11.3
ALT (8–45) U/L	5	Creatinine (0.45–0.82) md/dL	0.63
LDH (122–228) U/L	427	Na (138–146) mmol/L	139
Total bilirubin (0.3–1.3) mg/dL	1.95	K (3.6–5.1) mmol/L	4.1
Total protein (6.7–8.3) g/dL	4.8	Cl (99–108) mmol/L	106

Other tests such as thrombin time, reptilase time, PAI-1, as well as viscoelastic testing (TEG or ROTEM) were not performed. Abbreviations: CBC, complete blood count; WBC, white blood cell count; Hb, hemoglobin; MCV, mean corpuscular volume; CRP, C-reactive protein; AST, aspartate aminotransferase; ALT, alanine aminotransferase; LDH, lactate dehydrogenase; PT, prothrombin time; PT-INR, prothrombin time-international normalized ratio; APTT, activated partial thromboplastin time; BUN, blood urea nitrogen.

**Table 2:** A family of congenital hypofibrinogenemia.

Family Members	Age (Years)		Fibrinogen (mg/dL)		Sequencing (*FGB* Bβ Gene; exon 8)	Hemorrhagic Symptoms
			(Activity)	(Antigen)		
Father	35		99	97	p.403Try > stop	none
Mother	35		297	-	-	-
Elder sister	2		67	NT	NT	none
Proband (newborn)	0	Day 0	<50	NT	p.403Try > stop	none
		Day 8 *	61	67		

NT = not tested, * after FFP replacement.

## Data Availability

No additional data sets are associated with this paper.
